# Perceptions and attitudes of clinicians in Spain toward clinical practice guidelines and grading systems: a protocol for a qualitative study and a national survey

**DOI:** 10.1186/1472-6963-10-328

**Published:** 2010-12-03

**Authors:** Anna Kotzeva, Ivan Solà, José Miguel Carrasco, Petra Díaz del Campo, Francisco Javier Gracia, Enrique Calderón, Idoia de Gaminde, Maria Dolors Estrada, Flora Martínez, Carola Orrego, Rafael Rotaeche, Flavia Salcedo, Paola Velázquez, Pablo Alonso-Coello

**Affiliations:** 1 Catalan Agency for Health Technology Assessment and Research (CAHTA), Roc Boronat 81-95, Barcelona, Spain; 2 Iberoamerican Cochrane Centre, Clinical Epidemiology and Public Health Department, Institute of Biomedical Research (IIB Sant Pau). Barcelona, Spain; 3 Instituto Aragonés de Ciencias de la Salud, Zaragoza, Spain; 4 Agencia Laín Entralgo. Consejería de Sanidad y Consumo de la Comunidad de Madrid, Spain; 5 Instituto de Biomedicina de Sevilla. Virgen del Rocío University Hospital, Sevilla, Spain; 6 CIBER de Epidemiología y Salud Pública (CIBERESP), Spain; 7 Servicio de Docencia y Desarrollo Sanitarios. Departamento de Salud Gobierno de Navarra, Spain; 8 Unidad Docente de Medicina Familiar y Comunitaria de Sevilla. Sevilla, Spain; 9 Avedis Donabedian Institute, Universidad Autónoma de Barcelona, Barcelona, Spain; 10 Centro de Salud de Alza, Donostia-San Sebastián, Spain; 11 Consultas Médicas y Psicológicas, Barcelona, Spain

## Abstract

**Background:**

Clinical practice guidelines (CPGs) have become a very popular tool for decision making in healthcare. While there is some evidence that CPGs improve outcomes, there are numerous factors that influence their acceptability and use by healthcare providers. While evidence of clinicians' knowledge, perceptions and attitudes toward CPGs is extensive, results are still disperse and not conclusive. Our study will evaluate these issues in a large and representative sample of clinicians in Spain.

**Methods/Design:**

A mixed-method design combining qualitative and quantitative research techniques will evaluate general practitioners (GPs) and hospital-based specialists in Spain with the objective of exploring attitudes and perceptions about CPGs and evidence grading systems. The project will consist of two phases: during the first phase, group discussions will be carried out to gain insight into perceptions and attitudes of the participants, and during the second phase, this information will be completed by means of a survey, reaching a greater number of clinicians. We will explore these issues in GPs and hospital-based practitioners, with or without previous experience in guideline development.

**Discussion:**

Our study will identify and gain insight into the perceived problems and barriers of Spanish practitioners in relation to guideline knowledge and use. The study will also explore beliefs and attitudes of clinicians towards CPGs and evidence grading systems used to rate the quality of the evidence and the strength of recommendations. Our results will provide guidance to healthcare researchers and healthcare decision makers to improve the use of guidelines in Spain and elsewhere.

## Background

Clinical practice guidelines (CPGs) are designed to support decision-making processes in patient care and are classically defined as "systematically developed statements to assist practitioners' and patients' decisions about appropriate healthcare for specific circumstances" [1]. While there is some evidence that CPGs improve outcomes when they are appropriately implemented, [2,3] there are numerous factors which influence their acceptability and use by healthcare providers [4].

Some of the major barriers to guideline adherence can be related to practitioner, patient, organisational or guideline factors, [5-7] although practitioners appear to be a key factor. Aspects such as knowledge, perceptions of and attitudes toward CPGs in general, are professional-related. Other factors that affect the success of guideline implementation are content or format related, like clarity and presentation of the recommendations or the existence of a quick reference guide or algorithm [8]. The grading system used to classify the quality of the evidence and the strength of recommendations is an important characteristic of guideline format. However, the growing number of different grading systems employed across different institutions [9] could possibly be a source of confusion, impeding the correct comprehension of recommendations and their application in clinical practice.

The evidence on clinicians' knowledge, perceptions and attitudes toward CPGs is growing. The majority of published studies on this topic used quantitative design (surveys or record audits), investigating the association between barriers or facilitators and CPG use. Alternative approaches used by some investigators were qualitative designs, where concepts are collected in focus groups, interviews or through open-ended questions in questionnaires. Finally, some studies considered a mixed-method design including both qualitative and quantitative techniques.

Survey results were systematically reviewed in two publications, [4,10] concluding that most clinicians were supportive of CPGs, finding them to be useful, educational and likely to improve quality of care. Less frequent findings were that CPGs were impractical, unable to be used for individual patients, limited clinician autonomy, increased the likelihood of litigation or disciplinary action, and were used to cut costs [10].

A recent systematic review and meta-synthesis of qualitative studies of general practitioners' (GPs') attitudes to and experience with the use of CPGs [11] offers a conceptual framework for their interpretation. Six broad themes were identified in the analysis: questioning the guidelines; GPs' experience; preserving the doctor-patient relationship; professional responsibility; practical issues and guideline format. No systematic pattern in the distribution of these themes was found. However, comparative analysis and synthesis suggested that GPs' reasons for not following the CPGs differed according to whether the guidelines encouraged (prescriptive type) or discouraged (proscriptive type) particular interventions or behaviours [11].

Some authors suggest that clinicians find guideline contents too complex and confusing, and perceive difficulties in understanding the CPG development process. More specifically, there is very little research on clinicians' perceptions and understanding of the grading systems used to rate the evidence, quality, and strength of recommendations [12,13]. In our context, a qualitative study about the GRADE system showed important difficulties in understanding it and an important level of disagreement among the participating clinicians, with and without previous experience in CPG development [14]. However, the participants had hardly any experience with the use of this grading system, which requires substantial methodological expertise and practice.

Given this context there is a need for systematically reviewing the evidence on this topic and an assessment of the situation in our context. Additionally, the scarce information about the perceptions and understanding of the grading systems by healthcare professionals in the international literature provide the impetus for an investigation, both qualitative and quantitative, about this important aspect of clinical practice guidelines.

## Methods and design

### Goal and objectives

The main goal of this study is to explore the perceptions and attitudes of Spanish practitioners (GPs and hospital-based specialists, both with or without previous experience in guideline development) towards clinical practice guidelines and grading systems.

The specific objectives are to:

- Categorize, synthesize, and compare the perceptions of these different groups of healthcare professionals toward CPGs;

- Identify and describe the adoption/non-adoption factors specific to each group;

- Investigate whether different grading systems are better understood, and the influence of the wording of recommendations on understanding, acceptability and use of CPGs;

- Investigate whether different formats of CPGs influence acceptability and use of CPGs;

- Explore and compare the level of knowledge and understanding of different grading systems in these groups.

The design of this two-phase project will include qualitative and quantitative research techniques. During Phase I, with a qualitative design, group discussions will be carried out with the participation of GPs and hospital-based specialists, to explore in depth their perceptions and attitudes towards CPG and grading systems. In a posterior quantitative stage, Phase II, we will complement this information by means of a survey. The study has been approved by the Ethics Board of the Hospital de la Santa Creu i Sant Pau, Barcelona, Spain. 

### Phase I: Qualitative study

Qualitative research methods provide in-depth knowledge concerning perceptions, beliefs and values of the persons and groups involved and have proved to be extremely useful in the healthcare field [15,16]. These methods provide researchers with the opportunity to explore potential hypotheses on why participants hold the views they do. We thus considered this approach, and more specifically group discussions, as the most appropriate technique to gather information for our study objectives. The added value of group discussions [17,18] is that they permit us to explore dynamic interactions among group members in the light of certain cultural characteristics, concrete values, and references that may underlie individual preferences and shape the general position of the participants. The information gathered from group discussions will be analyzed following the sociological analysis disclosure model [19,20]. In this technique the reading and organization of data does not rely on the fragmentation of texts but on the interpretation of the different discursive positions of the participants and the main explanatory axes of their interventions [21].

#### 1. Group discussions structuring and participants

The basic topics to explore during the group discussions will be pre-selected by a group of experts (medical doctors, psychologists and sociologists with wide experience in CPGs development and implementation). These topics will be based on some of the most relevant and controversial issues around the development and implementation of CPGs in clinical practice, such as confidence, utility, knowledge and understanding of CPGs. Emphasis will be given to the importance of grading systems. Once these areas have been agreed upon, a semi-structured manual will be developed to ensure similarity in the moderation of the sessions.

A total of six groups are expected to be run: three groups of GPs (one group with, and two groups without previous experience in CPG development) and three groups of other hospital-based specialists (same distribution as GPs regarding previous experience). By "previous experience" in CPG development we will consider having participated in a guideline development group. Each group will consist of 8-9 clinicians, as generally recommended, [22] but not less than six. In these cases the sessions will be canceled and rescheduled. Participants will be selected taking into account their structural inter-group representation: medical specialty and years of experience, place of work, age and sex [18]. We will gather a purposefully selected sample, choosing participants according to their representativeness and capacity to provide rich and varied information in relation to the study objectives. On the other hand, the sample size will be considered sufficient when the information obtained has reached saturation, i.e., when information is repeated, redundant and provides no new aspects.

The group discussions will be carried out in four Spanish regions: Andalusia, Aragon, Catalonia and Madrid. We will recruit potential participants by means of a formal letter of invitation, or e-mail, and their travel costs will be reimbursed. Each group discussion will last approximately 60-90 minutes and will be recorded, with the consent of the participants.

#### 2. Moderation of group discussions

All investigators involved in group discussions will receive training in qualitative research methods, and on focus groups technique in particular. In order to achieve optimal standardization of the sessions, the previously developed semi-structured manual will be used. Still, its content might be a subject of constant adjustments depending on the findings that arise during the development of the group discussions [23].

An interviewer and a collaborator will be involved in moderating each group discussion. The role of the interviewer will be to lead the discussion by introducing the subjects of interest and managing the group dynamics so as to maintain focus and cover the programmed issues. We will take great care to avoid sharing opinions on the topic. The role of the collaborator will be to observe group dynamics and annotate any elements of the discussion, such as non-verbal language, responses to the interviewer's interventions, etc.

The interviewer will introduce the study objective indirectly, with the objective of analyzing the group's approach to the subject, and explain the need to record the session. Assurance of confidentiality will be provided to all group members, as well as of the independent nature of the study, explaining clearly to the potential participants that knowledge and quality of clinical practice will not be evaluated. Some basic demographic information will be collected before the beginning of each discussion group. Group sessions will be designed to be flexible and open to possible new topics and issues raised by participants. The two investigators will meet together after finishing the group meetings. This information will be analyzed in conjunction with the data obtained in the group discussions [23].

#### 3. Data organization, validation and analysis

All information generated will be audio-taped and stored digitally, thus representing the 'information corpus'. Audio-tapes will be transcribed literally and completed by the information collected by the interviewers and observers. To guarantee accuracy, transcriptions will be verified by the moderators of each group. For final data validation, the participants will be invited to read and comment on transcription contents [24].

Themes and patterns will then be identified and coded by two independent investigators, keeping in mind the texts as a whole and the contexts in which they have been produced. Each step in the configuration of the potential explicative axes requires new purposeful and iterative reading of texts to legitimate and corroborate the interpretation of findings [25]. Thus, to assure credibility, the data of the first group interviews will be re-assessed in the light of new findings. In the final stage of results interpretation the main explanatory axes will be organized and contrasted with the empirical reference of texts, in order to select the most representative verbatim statements. The investigators will develop a list of the identified concepts. The process and provisional analysis will be triangulated among investigators and, in case of divergence; consensus will be obtained through discussion.

### Phase II: Survey

#### 1. Questionnaire design

We will elaborate a single questionnaire divided into three general sections. The first part will collect information on the demographic characteristics and professional profile of participants (such as sex, age, qualifications and work experience). The second and third parts of the questionnaire will evaluate practitioners' knowledge, perceptions of and attitudes toward CPGs and grading systems. The information obtained in the group discussions (phase I) will be used as a starting point in the development of the questionnaire, providing insight into the social and symbolic representation of CPGs in our local circumstances. Previous to its application, the questionnaire will be piloted with a group of practitioners in order to modify or eliminate confusing and contradictory questions.

#### 2. Study sample

We have estimated a sample size of 600 respondents needed to answer one of the specific objectives of the survey (adequate comprehension of grading systems) in the GPs group, with a precision of 4%. To obtain this precision we dichotomized one item of the survey (adequate comprehension: yes/no) assuming maximum variability (50% of affirmative responses). A confidence interval of 95% will be applied to the percentage of affirmative responses.

#### 3. Recruitment of participants

Approximately 1200 clinicians working in Primary Care or Hospital Departments of the four regions participating in the study will be contacted. The questionnaire will be sent via e-mail to all potential participants in the two subgroups of interest (GPs and specialists) with a letter inviting them to collaborate and a link to an electronic version of the questionnaire. Three reminders will be sent to those who have not responded within two weeks. An investigator's contact details will be provided for participants to raise questions or doubts about the questionnaire or the project.

To potentiate practitioners' participation in the survey and to guarantee a high response rate, we will apply the following strategies which have proved to be successful: [26] the questionnaire will be concise and easy to complete (approximate duration 10-15 minutes); we will assure confidentiality of all personal data and will offer the participants the possibility to receive the final results of the survey.

#### 4. Analysis

For qualitative outcomes, between-group comparison will be described by means of contingency tables and inferences with the Chi Square Test or Fisher's Exact Test. Quantitative outcomes will be described using means and their corresponding standard deviations. Inferences will be made using an analysis of variance (ANOVA). If we encounter clearly defined application problems (abnormality and heteroscedasticity) the corresponding non-parametric test will be used. All analyses will be conducted using a bilateral approach. We will use the usual 5% significance level (α = 0.05) and the SPSS statistical package, version 11.5, to run the proposed analyses.

## Discussion

Clinicians' knowledge, perceptions and attitudes toward CPGs are important aspects towards the necessary implementation of these tools and subsequent change in clinical practice. To date, these aspects have been systematically reviewed in three main papers [4,10,11]. A number of later studies surveyed clinicians' knowledge about and attitudes towards CPG in general in different clinical settings: among general practitioners (GPs), [27-30] specialists, [28,31-34] and healthcare providers in Intensive Care Units [35-37]. Generally, most findings reported in these studies confirm that higher familiarity with the guidelines is related to more positive attitudes towards them and to more frequent reported use of CPGs.

Guidelines concerning both diagnosis and treatment of common diseases, developed on a national level and adapted locally, presented in summarized format and transmitted directly by the practitioners of the department, have the best chance of being used, as Riou et al. observed in a described setting [32]. On the other hand, the predictive role of characteristics, such as age, personality traits and professional qualifications, in professionals' attitudes towards CPGs is still controversial [31,36-38]. Additionally, several studies have focused on the factors which influence the use and acceptance of a specific guideline in a local context, [39-44] making the generalization of their results difficult.

Carlsen et al. [45] carried out focus groups amongst Norwegian GPs and add two important findings. First, the participants were concerned that guideline recommendations may be more heavily influenced by economic considerations than by clinical aspects. Second, in contrast to earlier findings, changes in recommendations due to evidence-based updates were mostly viewed positively. This latter point should be viewed in light of the Norwegian context, which has a relatively small number of published guidelines. In addition to these findings Boivin et al., using semi-structured focus group interviews, observed that in circumstances where clinicians judge patient participation in decision-making to be important, they perceive tension between the need to respect patients' preferences and the pressure to apply guidelines [46]. The authors conclude that CPGs should include information that supports shared decision-making in addition to their current focus on influencing prescription patterns, costs and health outcomes.

In Spain, to the best of our knowledge, this study will be the first to explore the perceptions, beliefs and attitudes of clinicians (GPs and other specialists, with or without previous experience in guideline development) towards CPGs. In relation to grading systems used to rate the evidence, quality and strength of recommendations, it will be one of the first to explore this issue qualitatively in more detail. Limitations and difficulties which we expect to face during the study originate from the natural complexity of qualitative designs and more specifically, group discussions. The researcher, or moderator, in these types of studies has less control over the data produced [47] than in either quantitative studies or in-depth interviews. The moderator has to allow participants to talk to each other, ask questions and express doubts and opinions, while having very little control over the interaction other than generally keeping participants focused on the topic. By its nature, the group discussion method is open-ended and cannot be entirely predetermined. Additionally, it should not be assumed that the individuals in a group discussion are expressing their own definitive individual view. They are speaking in a specific context, within a specific culture, and thus sometimes it may be difficult for the researcher to clearly identify an individual message. It is to be anticipated that dominant group members influence the statements and opinions of others, as they do in "real" group meetings and other social settings [45]. Nevertheless, there are also important advantages to carrying out group discussions. They allow for gathering a lot of relevant information in a short period of time, offering insight on the existing contradictions and differences in the individual opinions of the participants. This social interaction is produced in a real context and helps getting a broader conceptual perspective of the issues discussed. Another potential disadvantage of corpus analysis is that the findings are not necessarily valid in other settings and cannot be extended to wider populations. However, we will undertake our study in a wide and diverse population of clinicians, assuring wide applicability of our results in Spain and probably elsewhere.

The limitations would be potentially neutralized by completing initial results from group discussions with survey findings. On the other hand, the most apparent limitation of the survey could be a possible low response rate. However, in survey research, no single response rate is considered a standard [48,49]. For some surveys, a response rate of 80 percent is desired; in others, 60 percent is deemed adequate. Mail surveys typically have lower response rates than other types of surveys, and because non-response may introduce error, researchers should take steps designed to promote responses. Some of these steps include personally contacting potential respondents and asking them to participate, sending a reminder to non-respondents, assuring respondents of confidentiality, and making the survey short and easy to complete. In our study, all these measures will be adopted. Finally, caution will be taken when interpreting information about the connection between attitudes and actions. Research indicates that positive attitudes to guidelines do not necessarily mean that doctors follow their recommendations [10,50].

Based on the results from both phases of our study, we expect to deepen our understanding about the perceived difficulties and barriers to the comprehension and application of CPGs and to answer whether different grading systems, recommendation phrasing and guideline format presentation influence these processes. We expect to identify and describe the CPGs' adoption factors specific to each professional group participating and thus inform health professionals and decision makers about the needs and challenges in this field.

## Abbreviations

CPGs: Clinical practice guidelines; GPs: general practitioners; GRADE: Grading of Recommendations Assessment, Development and Evaluation; ANOVA: analysis of variance; SPSS: Statistical Package for the Social Sciences.

## Competing interests

The authors declare that they have no competing interests.

## Authors' contributions

PAC and IS conceptualized the study. AK and PAC drafted a first version of the protocol. All authors participated in revising it critically for important intellectual content and have given final approval of the version to be published.

## Pre-publication history

The pre-publication history for this paper can be accessed here:

http://www.biomedcentral.com/1472-6963/10/328/prepub
